# Parapharyngeal space carcinoma ex pleomorphic adenoma: case report and literature review

**DOI:** 10.1016/j.bjorl.2020.11.021

**Published:** 2021-01-02

**Authors:** İbrahim Yağcı, Seyhan Özakkoyunlu Hasçiçek, Metin Figen, Alican Çoktur, Mehmet Ece, Suat Turgut

**Affiliations:** a University of Health Sciences, Sisli Hamidiye Etfal Research and Training Hospital, Department of Otolaryngology & Head and Neck Surgery, Turkey; b University of Health Sciences, Sisli Hamidiye Etfal Research and Training Hospital, Pathology Department, Turkey; c University of Health Sciences, Sisli Hamidiye Etfal Research and Training Hospital, Radiation Oncology Department, Turkey

## Introduction

The parapharyngeal space (PPS) is a potential space filled with fibrofatty tissue, extending from the skull base to the level of the hyoid bone.

The space is divided into prestyloid and poststyloid compartments by the fascia extending from styloid process to tensor palatine. The frequency of tumors that may arise from each compartment can vary according to the characteristics of the tissues in these regions. Determining the localization may help the clinician in predicting the possible diagnosis of the tumor. Salivary gland tumors, lymphomas and lipomas are frequently detected in the anterior compartment, while nerve sheath tumors, vascular and soft tissue tumors are frequently located in the posterior compartment.^1^

PPS tumors are rare tumors. They constitutes 0.5% of head and neck neoplasms.^2^ Tumors originating from this region are frequently benign. The malignancy rate is about 20%–25% and most of them are salivary gland malignancies.^3^ Salivary gland tumors in this region generally originate from the adjacent parotid deep lobe, while to a lesser extent, tumors originating from minor salivary glands can also be detected.

Carcinoma ex pleomorphic adenoma (CXPA) is an aggressive and high-grade epithelial tumor that develops from the pleomorphic adenoma (PA). Although it often develops as recurrent PAs, it can also develop as a primary PA.

CXPA is a rare salivary gland tumor. It constitutes 3%–5% of all salivary gland neoplasms and 5%–15% of all salivary gland malignancies.^4^ Its incidence in the population is 0.63/1 million people. It is typically high grade and disease-related death is often be seen due to distant metastases. The most common location is the parotid gland; CXPA constitutes 5%–15% of primary parotid gland malignancies.

There are very few primary PPS CXPAs reported in the literature to date. The tumor detected in PPS can originate from parotid deep lobe tissues or PPS minor salivary glands. In this article, we present a case originating from PPS minor salivary gland, and a review of the literature.

The pathological examination of the case was evaluated by an experienced pathologist and classified according to the 4th edition of the World Health Organization classification of head and neck tumors. In histopathological analysis, tumor subtype and grade by immunohistochemical staining, infiltration of capsule and surrounding tissue, and carcinoma/PA tissue ratio were evaluated.

## Case report

**Figure 1:**
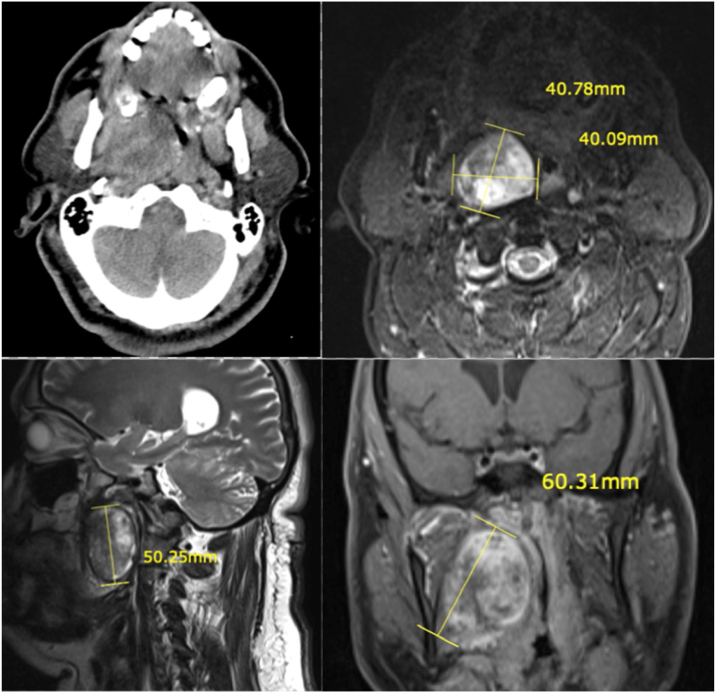
(a) Axial contrast-enhanced CT imaging. (b) Axial T2-weighted MRI. (c) Sagittal T2-weighted MRI. (d) Coronal plane T1-weighted fat suppressed and contrast-enhenced MRI.

A 50 year old male patient who complained about a recent increase of snoring and apnea attacks was admitted to our clinic with these symptoms. The patient, with known hypertension, obesity and OSAS diagnoses, did not have routine controls for these conditions. The patient also complained of swelling and difficulty swallowing. There was no mass on neck palpation. On the oropharyngeal examination, the soft palate and pharyngeal lateral wall mucosa displaced by the effect of the underlying swelling, resulting in upper airway obstruction. Contrast-enhanced magnetic resonance (MR) imaging showed a T1 hypointense, T2 hyperintense, 55 × 50 mm sized, well-circumscribed tumor (Fig. 1). These imaging findings supported the diagnosis of a pleomorphic adenoma. Transoral FNAB result was reported as suspicious cytology in terms of pleomorphic adenoma and malignancy.

**Figure 2:**
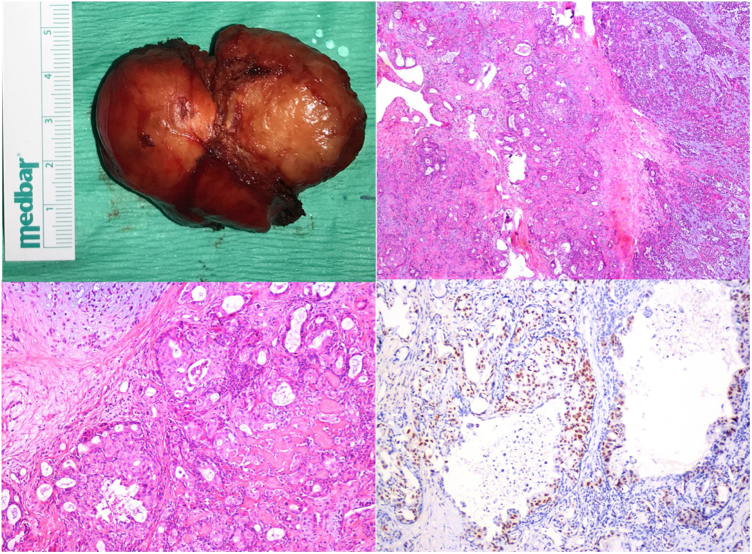
(a) Macroscopic view of the tumor. (b) Tumoral infiltrate progressing in the glandular pattern of the hyalinized stroma, adjacent to the pleomorphic adenoma parenchyma containing the chondromyxoid matrix (H&E, ×10). (c) Cribriform glands containing areas of comedo necrosis in their lumen (H&E, ×10). (d) Nuclear Androgen receptor staining in tumor cells (×20).

The patient was operated under general anesthesia with a transcervical approach and the tumor was totally excised. The pathology examination was reported as minimally invasive carcinoma ex pleomorphic adenoma (salivary duct carcinoma type). In immunohistochemical staining, CK7, p63 and androgen receptor were reactive. No staining was observed with CD117 and S100. The Ki67 index was reported as 30%–40% (Fig. 2). Capsular invasion was present in an area of 1 mm on the tumor; the CXPA/PA ratio was 61.5%.

Following surgery, patient underwent an adjuvant 60 Gy of external radiotherapy due to the pathological features of the tumor.

There was no locoregional or distant recurrence in MR imaging and positron emission tomography—computed tomography (PET/CT) 6 months after the treatment. The patient was followed up in the first year without disease.

## Discussion

Primary PPS malignancies are rare tumors. In addition, CXPA is a rare tumor among salivary gland malignancies. The coexistence of both conditions is very rare and there are very few reported cases.

In a review of the last 50 years of literature, 2153 parapharyngeal tumors were detected.^5^ CXPA was detected in only 28 patients among 451 malignant tumors originating from the parapharyngeal region. Among all patients, CXPA accounted for 6.2% of all malignant PPSTs and 1.3% of all PPSTs. When the publications in the English language that were published after this review were re-scanned, 3 more case reports with primary parapharyngeal space CXPA were detected.^2^, ^6^ Thus, the case we present in this article is the 32nd primary PPS CXPA case in the literature.

According to many studies, the sensitivity of FNAB in the diagnosis of CXPA is 64%–92% and its specificity is 86%–98%. In a study in which 24 patients with CXPA were retrospectively analyzed, only 43% of 16 patients with FNAB were diagnosed preoperatively as malignancy.^7^ Patients who could not be diagnosed with malignancy preoperatively were frequently misdiagnosed as PA. The probable reason for this situation is that no sample was taken from the CXPA focus within the tumor, that is, insufficient sampling. Frozen section pathological examination can be selected in many surgical procedures in the head and neck region. False negative rate of frozen section evaluation in salivary gland malignancies is reported to be 9%–24%.

The preoperative FNAB result in our case was reported as “suspicious for malignancy”. There were findings suggestive of pleomorphic adenoma radiologically and there were no signs of invasion into the surrounding structures. Since the surgical technique will not change and no pathological lymphadenopathy was observed during the operation, frozen section examination was not performed in our case.

Histopathologically, the most common subtype of the tumor is intraductal carcinoma and the transition from PA to CXPA may be in the form of a malignant transformation. In the presented patient, the subtype of the tumor was high grade salivary duct carcinoma.

CXPA is divided into 3 subgroups in terms of presence of capsule invasion in pathological examination: intracapsular carcinoma (non-invasive/insitu), minimally invasive carcinoma (less than 1.5 mm capsule invasion) and invasive carcinoma (more than 1.5 mm capsule invasion). In a study evaluating 19 cases with PPS CXPA, tumor was intracapsular in 5 patients and minimally invasive in 7 patients.^7^ Orsen detected invasive tumors in 92% of patients.^8^ In another study, disease-related death was not observed in patients with less than 0.5 cm invasion and noninvasive tumors.^9^ As the tumor invasion size increased, the risk of recurrence was increased. In the presented patient, 1 mm capsule invasion was detected, and the tumor was reported as minimally invasive CXPA.

When the CXPA/PA ratio is calculated in size; Orsen observed that this rate was more than 50% in 80% of patients.^8^ Zbaren et al. reported more than 66% in 42% of patients.^7^ Lewis et al. found that this rate was more than 50% in 84% of patients.^9^ In the patient we presented, we calculated the CXPA/PA ratio as 61.5%.

In a study in which 24 patients were evaluated and recurrence was observed in 6 patients, 5 year survival was 76%.^7^ The high survival rates in this study have been associated with the high percentage of patients with early stage and noninvasive tumors, and additionally with the administration of adjuvant radiotherapy (RT) to half of the operated patients. In another study, the 2 and 5 year overall survival rates of patients diagnosed with CXPA were 84.6% and 68.5%; disease specific survivals were 90.3% and 80.4%.^10^

Increased tumor size was associated with poor survival. It may be necessary to give adjuvant RT according to tumor capsule invasion, pathological subtype, and pathological grade. Thus, it is possible to increase the local control rates of the disease. Elective therapy for neck (neck dissection or RT) is recommended for high-grade CXPA. However, the effect of RT on survival is limited, especially due to the high risk of distant metastases of high-grade tumors. No positive effect of adding adjuvant CT to treatment has been demonstrated. The presented patient received elective RT for both local and neck due to the tumor's high-grade histology and minimal capsule invasion.

In the majority of patients, transcervical or transcervical-transparotid approaches can be performed as surgical techniques. These approaches provide direct visualization of the tumor and neurovascular structures, and also have acceptable cosmetic results. The external approach can also be performed with an endoscope assisted technique, especially in lesions extending to the base of the skull. In contrast, transoral surgery or transoral robotic surgical techniques are not recommended, especially due to the risk of tumor disintegration in surgery of malignant tumors, or because of the risk of injury to neurovascular structures in the parapharyngeal region. Intraoral tumor resection can be performed in small lesions, but there may be difficulties in preserving PPS neurovascular structures and the risk of recurrence associated with tumor disintegration should not be forgotten. For this reason, the transcervical approach will be more suitable, especially in cases of PPS tumor diagnosed with malignancy. In addition, transcervical-transparotid approach should definitely be preferred for the visualization and protection of the facial nerve in parotid deep lobe lesions. It may be necessary to use the mandibular swing approach in large-sized or vascular lesions that cannot provide adequate exposure.

Due to the slow growth pattern and the tumor located in a hidden area such as the parapharyngeal region, PAs from the deep lobe or PPS can reach large sizes without symptoms or with mild symptoms for many years. For this reason, transcervical-transparotid surgery, including defining the facial nerve, is safer especially in the treatment of PPS tumors originating from the parotid deep lobe.

Parotid deep lobe tumors can be benign or malignant. Parotid gland malignant tumors (whether superficial or deep lobe) require not only the excision of the main mass but also the resection of the entire parotid gland tissue as total parotidectomy. In the presented case, parotidectomy or facial nerve identification was not performed because of the presence of intact adipose tissue between the deep lobe of the parotid and the mass, and thus the mass originating from the parapharyngeal region.

## Conclusion

It is known that the longer pleomorphic adenoma is left untreated, the risk of developing malignancy increases. In the presented patient, we believe that the tumor in the parapharyngeal region had grown enough to form upper airway obstruction, and that the tumor developed on a PA that has remained untreated for a long time.

## Conflicts of interest

The authors declare no conflicts of interest.
